# Emerging role of circular RNAs in the pathogenesis of ovarian cancer

**DOI:** 10.1186/s12935-022-02602-1

**Published:** 2022-04-29

**Authors:** Soudeh Ghafouri-Fard, Tayyebeh Khoshbakht, Bashdar Mahmud Hussen, Mohammad Taheri, Majid Samsami

**Affiliations:** 1 Department of Medical Genetics, School of Medicine, Shahid Beheshti University of Medical Sciences, Tehran, Iran; 2 Urology and Nephrology Research Center, Shahid Beheshti University of Medical Sciences, Tehran, Iran; 3 Department of Pharmacognosy, College of Pharmacy, Hawler Medical University, Arbīl, Kurdistan Region Iraq; 4 Center of Research and Strategic Studies, Lebanese French University, Arbīl, Kurdistan Region Iraq; 5 Institute of Human Genetics, Jena University Hospital, Jena, Germany; 6 Cancer Research Center, Shahid Beheshti University of Medical Sciences, Tehran, Iran

**Keywords:** circRNA, Ovarian cancer, Biomarker, Expression

## Abstract

Ovarian cancer is a female malignancy with high fatality-to-case ratio, which is due to late detection of cancer. Understanding the molecular mechanisms participating in these processes would facilitate design of therapeutic modalities and identification of novel tumor markers. Recent investigations have shown contribution of circular RNAs (circRNAs) in the evolution of ovarian cancer. These transcripts are produced through a back-splicing mechanism. The enclosed configuration of circRNAs protects them from degradation and potentiates them as biomarkers. Several circRNAs such as circMUC16, circRNA_MYLK, circRNA-UBAP2, circWHSC1, hsa_circ_0013958, circFGFR3, hsa_circRNA_102958 and circ_0072995 have been found to be up-regulated in this cancer, acting as oncogenes. On the other hand, circ-ITCH, circPLEKHM3, circ_100395, circ_0078607, circATRNL1, circHIPK3, circRHOBTB3, circEXOC6B, circ9119 and CDR1as are among down-regulated circRNAs in ovarian cancer. Expression levels of circCELSR1, circ_CELSR1, circATL2, circNRIP1, circTNPO3 and hsa_circ_0000714 have been shown to affect resistance of ovarian cancer cells to chemotherapy. Moreover, circ_100395, circFGFR3, circ_0000554, circCELSR1, circ-PTK2, circLNPEP, circ-CSPP1, circ_0000745, circ_100395 and circPLEKHM3 have been shown to regulate epithelial-mesenchymal transition and metastatic ability of ovarian cancer cells. In the current review, we explain the roles of circRNAs in the evolution and progression of ovarian cancer.

## Introduction

Epithelial ovarian cancer is the most fatal kind of malignancy among females [1]. Early detection of ovarian cancer is hindered by the lack of suitable tumor biomarkers, thus disease is usually diagnosed in advanced stages. Due to late diagnosis, this malignancy has the highest fatality-to-case ratio among gynecological cancers [2]. Malignant progression and prompt development of drug resistance are other problems encountered in clinical management of ovarian cancer [3]. The vast majority of ovarian tumors originate from the epithelial surface of the ovary. Others arise from germ cells or stromal cells. The main subclasses of epithelial cancers are serous, endometrioid, mucinous, clear cell, and undifferentiated cancers. These subclasses have different risk factors, clinical behaviors, and treatment responses [3]. From a molecular point of view, both genetic alterations in epithelial cells and reprogramming of the tumor microenvironment contribute in the evolution of ovarian cancer [3]. Understanding the molecular mechanisms participating in these processes would facilitate design of therapeutic modalities and identification of novel tumor markers [4, 5].

Circular RNAs (circRNAs) are a group of non-coding RNAs with a covalently closed configuration [6]. These transcripts have been initially regarded as a splicing error. However, their roles in the regulation of gene expression have been recognized during recent years. These transcripts are produced through back-splicing or exon skipping of precursor mRNAs [7]. These evolutionarily conserved transcripts have a high abundance in the cytoplasm and are more stable than linear transcripts. They can regulate expression of parental genes, modulate alternative splicing events or mRNA translation and act as molecular sponges for miRNAs or RNA-binding proteins. Moreover, they can occasionally produce peptides or proteins [7]. Recent studies have shown contribution of circRNAs in the pathogenesis of cancers [8]. In the current review, we explain the roles of circRNAs in the evolution and progression of ovarian cancer.

### Up-regulated circRNAs in ovarian cancer

CircMUC16 is among up-regulated circRNAs in ovarian cancer tissues whose up-regulation in these tissues has been correlated with higher stage and grade. Down-regulation of circMUC16 in ovarian cancer cells has inhibited autophagy flux, while its forced over-expression has increased autophagy flux of cells. The impact of circMUC16 on autophagy has been shown to enhance invasion and metastasis of ovarian cancer cells. This effect has been exerted through binding to miR-199a-5p and releasing Beclin1 and RUNX1 from its suppressive roles. Moreover, RUNX1 has been found to elevate circMUC16 levels through increasing its transcription. Notably, circMUC16 can also directly bind to ATG13 and enhance its expression [9].

circRNA_MYLK is another up-regulated circRNAs in ovarian cancer tissues. Patients with over-expression of circRNA_MYLK have been found to have a more advanced stage and a lower overall survival time. In vitro studies have shown that circRNA_MYLK silencing attenuates proliferation ability of cells. Functionally, circRNA_MYLK can enhance the malignant progression of ovarian cancer cells through regulation of miR-652 levels [10].

Besides, circRNA-UBAP2 has been shown to be up-regulated this type of cancer. CircRNA-UBAP2 silencing has suppressed proliferation of ovarian cancer cells and induced their apoptosis. Mechanistically, circRNA-UBAP2 can target miR-382-5p and down-regulate its expression to release PRPF8 from its inhibitory effects [11].

**Fig. 1:**
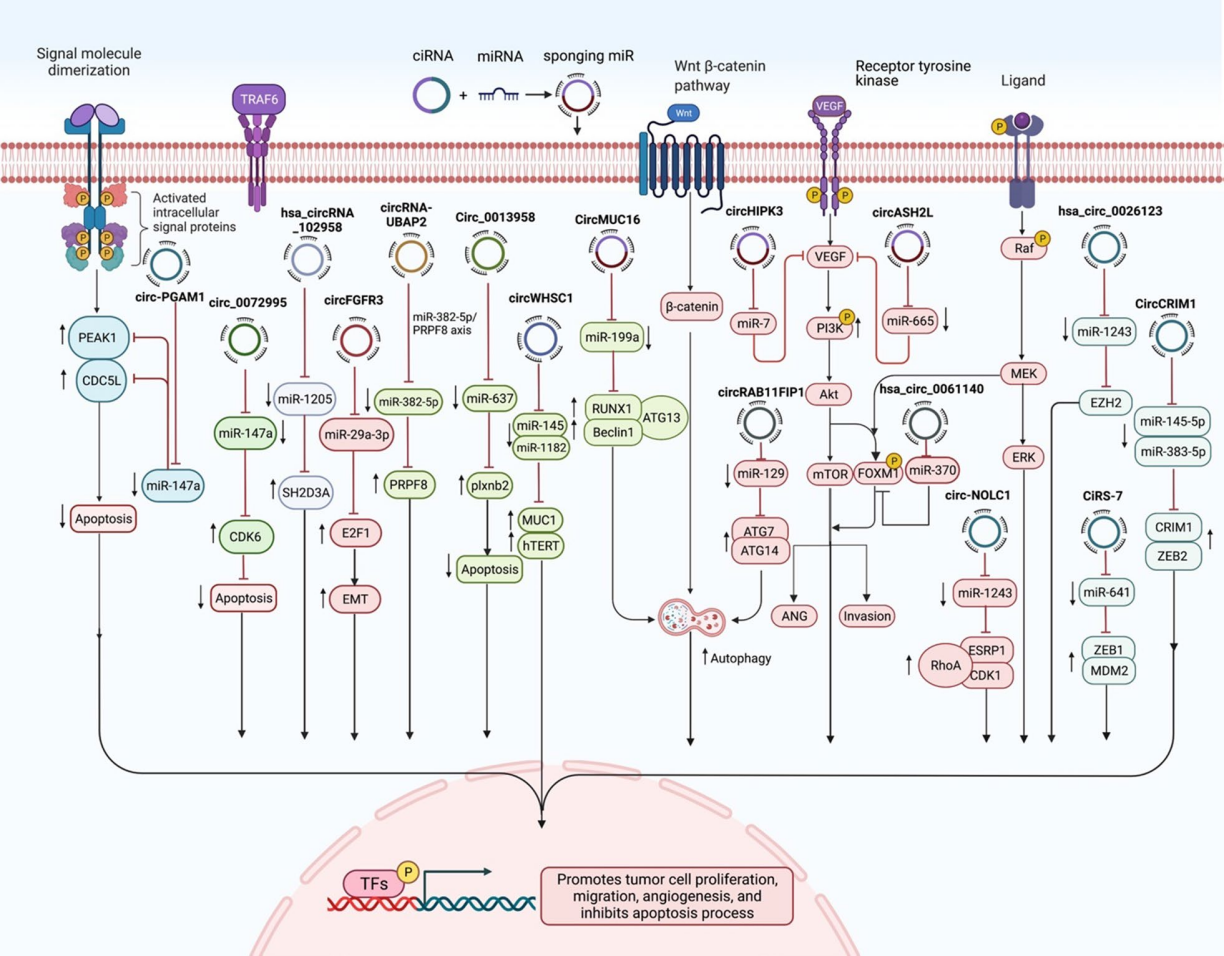
A schematic representation of the effects of some oncogenic circRNAs in the progression of ovarian cancer. These circRNAs can sponge tumor suppressor miRNAs such as miR-147a, miR-1205, miR-29a, miR-382, miR-637, miR-145, miR-1182, miR-129 and miR-1243, thus increasing expression of certain oncogenes that affect activity of cancer-related signaling pathways

CircUBAP2 is another circRNA whose over-expression in ovarian cancer tissues has been correlated with clinical stage and survival of patients. This circRNA is mainly located in the cytoplasm. Up-regulation of circUBAP2 could enhance proliferative and migratory capacities of ovarian cancer cells. This circRNA acts as a sponge for miR-144 to release CHD2 from its inhibitory effects [12]. Figure 1 shows the effects of some oncogenic circRNAs in the progression of ovarian cancer.

In order to find the impact of circRNAs in autophagy, Zhang et al. have assessed expression profile of circRNAs, miRNAs, and mRNAs in ovarian cancer cells after induction with Torin 1. They have reported up-regulation of 504 circRNAs and down-regulation of 478 ones. CircRAB11FIP1 has been among differentially expressed circRNAs. Expression of this circRNA has been found to be higher in epithelial ovarian cancer samples compared with normal ovarian tissues. Its silencing has suppressed the autophagic flux of SKOV3 cells. CircRAB11FIP1 has been shown to directly bind to miR-129 and regulate expression of miR-129 targets ATG7 and ATG14. CircRAB11FIP1 could also bind with DSC1 to assist its interaction with ATG101 [13]. Table 1 summarizes the results of studies that reported up-regulation of circRNAs in ovarian cancer.

**Table 1:** Up-regulated circRNAs and Ovarian cancer

circRNA	Clinical samples/animal model	Assessed cell lines	Targets/regulators/signaling pathways	Description	References
CircMUC16	3 EOC tissues and 4 healthy ovarian tissues/6-week-old BALB/c nude mice	SKOV3, ES-2, A2780 and CAOV-3	miR-199a-5p, Beclin1, RUNX1 and ATG13	↑↑ circMUC16: ↑ autophagy flux of SKOV3 cells ↑ autophagy flux of SKOV3 cells: ↑ EOC invasion and metastasis	[9]
circRNA_MYLK	46 pairs of tumor tissues and ANCTs	SKOV3, OVCAR3, PEO1, 3AO, A2780, CAOV3 and HOSEPiCs	miR-652	Patients with high levels of circRNA_MYLK showed a higher pathological staging and a lower OS rate ∆ circRNA_MYLK: ↓ cell proliferation	[10]
circRNA-UBAP2	20 pairs of tumor tissues and ANCTs	SKOV3, No. KL; OVCAR-3, No. H-OVCAR-3; ES-2, No. H-ES-2 and A2780, No. H-A2780 and IOSE80	miR-382-5p/PRPF8 axis	↑↑ circRNA-UBAP2: ↑ proliferation and ↓ apoptosis via sponging miR-382	[11]
circRNA-UBAP2	24 pairs of tumor tissues and ANCTs	A2780, HEY, OVCAR3, HO8910, SKOV3 and IOSE	miR-144	Expression of circRNA-UBAP2 was negatively associated with TMN stage and five-year survival of OC patients ↑↑ circRNA-UBAP2: ↑ proliferation and migration via sponging miR-144	[12]
circWHSC1	79 patients and 13 healthy controls/4-week-old female BALB/c nude mice	CAOV3 and OVCAR3	miR-145, miR-1182, MUC1 and hTERT	↑↑ circWHSC1: ↑ cell proliferation, migration and invasion, and ↓ cell apoptosis via sponging miR-145 and miR-1182	[14]
Hsa_circ_0013958	45 pairs of tumor tissues and ANCTs	A2780, OVCAR‐3 and HOSEpiC	–	High levels of Hsa_circ_0013958 were related to patient FIGO stage and lymph node metastasis ∆ hsa_circ_0013958: ↓ proliferation, migration, and invasion and ↑ apoptosis	[15]
Circ_0013958	30 pairs of tumor tissues and ANCTs/5-week-old female BALB/c nude mice	HOSE, SKOV3 and CAOV3	miR-637/PLXNB2 axis	∆ circ_0013958: ↓ proliferation, migration, invasion, and ↑ apoptosis	[16]
circFGFR3	35 pairs of tumor tissues and ANCTs	OSE, SKOV3, A2780, OV2008 and IGROV1	miR-29a-3p/E2F1 axis	↑↑ circFGFR3: ↑ EMT process	[17]
hsa_circRNA_102958	41 pairs of tumor tissues and ANCTs	OC cell lines and IOSE80 cells	miR-1205/SH2D3A axis	∆ hsa_circRNA_102958: ↓ proliferation, migration and invasion	[18]
circ_0072995	40 pairs of tumor tissues and ANCTs/4 to 6-week-old BALB/c nude mice	HO8910, A2780, and IOSE80	miR-147a/CDK6 axis	↑↑ circ_0072995: ↑ cell proliferation, migration and ↓ apoptosis	[19]
circ_0072995	47 pairs of tumor tissues and ANCTs/6-week-old female BALB/c nude mice	IOSE-80, OVCAR-3 and SK-OV-3	miR-122-5p/SLC1A5 axis	∆ circ_0072995: ↓ cell proliferation, migration, invasion and ↑ cell apoptosis	[20]
circEPSTI1	50 pairs of tumor tissues and ANCTs/4‐week‐old BALB/c nude mice	–	miR-942/EPSTI1 axis	∆ circEPSTI1: ↓ cell proliferation, invasion and ↑ apoptosis	[21]
circ-LOPD2	normal ovarian tissue, benign tumor, borderline tumor and ovarian cancer tissues	CAOV3, A2780 and OVCAR3	miR-378	∆ circ-LOPD2: ↓ cell growth	[22]
circGFRA1	50 pairs of tumor tissues and ANCTs/4-week old BALB/c nude mice	OV119 and A2780	miR-449a/ GFRA1 axis	∆ circGFRA1: ↓ cell proliferation and invasion and ↑ apoptosis	[23]
circ0004390	10 ovarian cancer tissues and 6 normal ovarian tissues	KOV3, HeyA8, OVCAR429 and HEK- 293 T	miR-198/MET axis	∆ circ0004390: ↓ proliferation	[24]
circKIF4A	50 pairs of tumor tissues and ANCTs/4-week-old female BALB/c nude mice	CAOV3 and SKOV3	miR-127/ JAM3 axis	∆ circKIF4A: ↓ cell proliferation and migration	[25]
circ_0000554	–	HO8910	miR-567	↑↑ circ_0000554: ↑ cell growth, invasion, and EMT process	[26]
circKRT7	5-week-old BALB/c mice	SKOV3, ES-2, CoC1, Caov-3, and Caov-4	miR-29a-3p/COL1A1 axis	∆ circKRT7: ↓ cell proliferation, migration and invasion via sponging miR-29a-3p	[27]
circCELSR1	4-week-old female athymic nude mice	SKOV3, A2780, IGROV1, CAOV3, and IOSE80	miR-598/ BRD4 axis	∆ circCELSR1: ↓ proliferation, migration, invasion and EMT process and ↑ apoptosis	[28]
circCELSR1	36 pairs of tumor tissues and ANCTs/6-week-old female BALB/c athymic nude mice	SKOV3, HeyA-8 and IOSE-80	miR-1252/FOXR2 axis	∆ circCELSR1: ↓ cell growth, ↑ G0/G1 arrest and apoptosis	[29]
circCELSR1	–	–	miR-149-5p/SIK2 axis	∆ circCELSR1: ↓ viability, colony formation and cell cycle process, ↑ paclitaxel sensitivity and cell apoptosis	[30]
circHIPK3	69 pairs of tumor tissues and ANCTs	A2780, HO8910, SKOV3, CAOV3 and HOEC	–	Patients with higher levels of circHIPK3 showed lymph node invasion, FIGO stage, and worse DFS and OS	[31]
circHIPK3	66 pairs of tumor tissues and ANCTs	SKOV3	miR-7/VEGF axis	∆ circHIPK3: ↓ tumorigenicity of ovarian cancer cells, proliferation and **↑** apoptosis	[32]
circRAB11FIP1	ovarian cancer tissues and serum samples from 70 EOC and 30 matched non-carcinoma tissue samples/6-week-old BALB/c nude mice	SKOV3	miR-129/ ATG7 and ATG14	∆ circRAB11FIP1: ↓ autophagic flux of ovarian cancer SKOV3 cells ↑↑ circRAB11FIP1: ↑ autophagy, proliferation and invasion	[13]
Hsa_circ_0009910	50 pairs of tumor tissues and ANCTs	SKOV3	miR-145	Hsa_circ_0009910 induces proliferative and motile phenotypes via sponging miR-145 in ovarian cancer cells	[33]
circASH2L	50 pairs of tumor tissues and ANCTs/4-week-old female athymic BALB/c nude mice	A2780, TOV112D, OVCAR-3, SKOV3 and ISOE80	miR-665/VEGFA axis	∆ circASH2L: ↓ invasion and cell growth in vitro, angiogenesis and lymphangiogenesis in vivo	[34]
circ-PGAM1	15 EOC tissues and 15 normal ovary tissues/nude mice	CAOV3, SKOV3, OVCAR3, ES‐2 and 293T cells	miR-542-3p/CDC5L/PEAK1 pathway	∆ circ-PAGM1: ↓ proliferation, migration, and invasion of ovarian cancer cells and ↑ apoptosis	[35]
circRhoC	127 ovarian cancer tissues and 24 normal ovarian tissues	A2780 cells	miR-302e/ VEGFA axis	↑↑ circRhoC: ↑cell viability, migration and invasion via sponging miR-302e	[36]
circPUM1	62 EOC and 13 normal ovarian tissues/5-week-old female BALB/c nude mice	A2780, CAOV3 and HMrSV5	miR-615-5p, miR-6753-5p, NF-κB and MMP2	↑↑ circPUM1: ↑ proliferation, migration, and invasion and ↓ apoptosis CircPUM1 showed to act on the peritoneum and increase metastasis of cancer in the form of cancer-derived exosomes	[37]
circ_0007841	43 pairs of tumor tissues and ANCTs/6-week-old BALB/c nude mice	SKOV3, OVCAR3 and IOSE80	miR-151-3p/MEX3C axis	∆ circ_0007841: ↓ proliferation, migration and invasion	[38]
hsa_circ_0026123	20 pairs of tumor tissues and ANCTs/4 weeks old female BALB/c nude mice	A2780, TOV112D, SKOV3, OVCAR-3 and ISOE80	miR-124-3p/EZH2 axis	∆ hsa_circ_0026123: ↓ proliferation and metastasis	[39]
circFoxp1	112 EOC patients and 82 healthy controls/nude mice	COC1, OVCAR3, SKOV3, SKOV3/DDP and IOSE-80	miR-22, miR-150-3p, CEBPG and FMNL3	↑↑ circFoxp1: ↑ proliferation and DDP resistance High levels of circFoxp1 were correlated with lymphatic metastasis, distant metastasis, FIGO stage, primary tumor size, residual tumor diameter, and clinical response	[40]
CircCRIM1	130 ovarian cancer tissues and 24 normal ovarian tissues/4-week-old female BALB/c nude mice	OVCAR3 and CAOV3	miR-145-5p, miR-383-5p, CRIM1 and ZEB2	↑↑ CircCRIM1: ↑ cancer progression in vitro and tumor growth in vivo	[41]
hsa_circ_0061140	4-week-old male BALB/c nude mice	SKOV3 and A2780	miR-370/FOXM1 axis	∆ hsa_circ_0061140: ↓ proliferation and migration	[42]
circ_0061140	20 PTX-resistant human ovarian cancer tissues and 19 PTX-sensitive human ovarian cancer tissues/5-week-old female BALB/c nude mice	SKOV3, HeyA8 and IOSE-80	miR-136/CBX2 axis	∆ circ_0061140: ↓ proliferation, migration and invasion, and ↑ apoptosis and PTX sensitivity	[43]
circ-PVT1	GTEx database	SKOV3 and A2780	miR-149-5p/FOXM1 axis	∆ circ-PVT1: ↓ proliferation, migration and invasion High levels of PVT1 were correlated with shorter OS in OV patients	[44]
circ-PVT1	–	CAOV3, SKOV3, SNU119, OVCAR3 and HOSEpiC	miR-149	∆ circ-PVT1: ↓ proliferation and ↑ apoptosis ↑↑ circ-PVT1: ↑ proliferation and ↓ apoptosis	[45]
circ_0015756	55 pairs of tumor tissues and ANCTs/5-week-old female BALB/c nude mice	OV90, SKOV3 and IOSE80	miR-942-5p/CUL4B axis	∆ circ_0015756: ↓ proliferation, migration and invasion and ↑ apoptosis	[46]
circ_0025033	39 pairs of tumor tissues and ANCTs/6–8 weeks old female BALB/c mice	KOV3 and A2780 and IOSE80	miR-184/LSM4 axis	∆ circ_0025033: ↓ colony formation, migration, invasion and glycolysis metabolism	[47]
circ_0005276	49 pairs of tumor tissues and ANCTs	CAOV3 and SKOV3	ADAM9	∆ circ_0005276: ↓ migration High levels of circ_0005276 were associated with lymphatic metastasis and distant metastasis in EOC patients	[48]
circ-NOLC1	118 ovarian cancer tissues, 11 borderline tumor tissues, 11 benign ovarian tissues, 15 normal ovarian tissues/5-week-old female BALB/c nude mice	A2780, CAOV3, ES-2, HO8910, OVCAR3, and SKOV3	ESRP1, CDK1 and RhoA	↑↑ circ-NOLC1: ↑ proliferation, migration, and invasion tumor growth by binding ESRP1 and modulating CDK1 and RhoA expression	[49]
circBIRC6	–	SKOV3, SKOV3/DDP	miR-367-3p	∆ circBIRC6: ↓ proliferation of ovarian cancer cisplatin-resistant cells and ↑ apoptosis	[50]
circ-0001068	20 pairs of tumor tissues and ANCTs 95 OC patients and 53 healthy controls	–	miR-28-5p	Circ-0001068 was found to be delivered into T cells and induced PD1 expression by sponging miR-28-5p	[51]
circRNA051239	30 EOC patients and 10 healthy controls	SKOV3.ip, SKOV3, A2780, CAOV3 and OVCAR3	miR-509-5p/PRSS3 axis	∆ circRNA051239: ↓ proliferation and migration	[52]
circVPS13C	40 pairs of tumor tissues and ANCTs	A2780, SKOV3 and IOSE-80	miR-145 and MEK/ERK signaling	Propofol treatment: ↓ circVPS13C levels and ↑ miR-145 levels, thus ↓ viability, cell cycle and motility and ↑ apoptosis	[53]
circANKRD12	–	PA-1, SKOV3, Caov3, NIH:OVCAR-3 and APOCC	–	Downregulation of circANKRD12 compelled a strong phenotypic change in cell cycle, invasion and migration and metabolism in cancer cells	[54]
VPS13C-has-circ-001567	20 pairs of tumor tissues and ANCTs	SKOV3 and OV-1063	–	∆ VPS13C-has-circ-001567: ↓ proliferation, tumorigenicity and ↑ apoptosis High levels of VPS13C-has-circ-001567 were associated with tumor node metastasis stage and lymph node metastasis	[55]
circPIP5K1A	25 pairs of tumor tissues and ANCTs/4-week-old BALB/c nude mice	VCAR5, SKOV3, A2780, OV2008 and HCerEpiC	miR-661/IGFBP5 axis	∆ circPIP5K1A: ↓ proliferation, migration and invasion	[56]
circATL2	–	PTX-resistant OC tissues and cells	miR-506-3p/NFIB axis	∆ circATL2: ↓ colony formation, resistance of OC to PTX and ↑ cell cycle arrest and apoptosis in PTX-resistant OC cells	[57]
hsa_circ_0004712	30 pairs of tumor tissues and ANCTs/4–6 weeks old female BALB/c mice	OVCAR-3, SKOV-3 and IOSE-80	miR-331-3p/FZD4 axis	∆ hsa_circ_0004712: ↓ proliferation, colony formation, invasion and migration, and ↑ apoptosis	[58]
CiRS-7	40 pairs of tumor tissues and ANCTs/4-week-old male BALB/c nude mice	SKOV3, A2780, OV2008, IGROV1, ES-2 and HOSE	miR-641/ZEB1 or miR-641/MDM2 axis	∆ CiRS-7: ↓ cell growth and metastasis High levels of CiRS-7 were correlated with the TNM stages, lymph node metastasis status and overall survival rate in OC patients	[59]
circ-PTK2	26 ovarian cancer tissues and 11 normal ovary tissues/4–5-week-old female nude mice	SK-OV-3 and OVCAR-3	miR-639/FOXC1 axis	↑↑ circ-PTK2: ↑tumor formation, migration and invasion and EMT process	[60]
circLNPEP	40 pairs of tumor tissues and ANCTs/4-week-old male BALB/c nude mice	A2780, SKOV-3, OVCAR3, SK-BR-3, OV-56 and TOV-21 G	miR-876-3p/WNT5A axis	∆ circLNPEP: ↓ cell viability, proliferation, migration, invasion, angiogenesis, and EMT process and ↑ apoptosis	[61]
circNRIP1	56 pairs of tumor tissues and ANCTs/BALB/c nude mice	HOEC, A2780, SKOV3 and A2780/PTX and SKOV3/PTX	miR-211-5p/HOXC8 axis	∆ circNRIP1: ↓ PTX resistance of OC cells in vitro and OC tumor in vivo	[62]
circTNPO3	48 pairs of tumor tissues and ANCTs/nude mice	SKOV3, HeyA-8 and IOSE-80	miR-1299/NEK2 axis	∆ circTNPO3: ↑ sensitivity to PTX via promoting PTX-induced apoptosis in vitro and in vivo	[63]
hsa_circ_0051240	10 pairs of tumor tissues and ANCTs/6-week-old male nude mice	CAOV-3, SKOV-3, OVCAR-3 and H8910 and HOSE	miR-637/KLK4 axis	∆ hsa_circ_0051240: ↓ cell proliferation, migration and invasion in vitro, and tumor formation in vivo	[64]
circSETDB1	73 pairs of tumor tissues and ANCTs/4–6-week-old female BALB/c nude mice	A2780, SKOV3, IOSE-80 and 293 T cells	miR-129-3p/MAP3K3 pathway	∆ circSETDB1: ↓ cell proliferation, migration, invasion and ↑ apoptosis	[65]
circSETDB1	60 SOC patients [18 primary chemoresistance, 42 primary chemosensitive] and 60 healthy controls	–	–	High levels of circSETDB1 Were correlated with advanced clinical stage, lymph node metastasis and a shorter PFS time of SOC patients	[66]
hsa_circ_0000714	–	SKOV3, A2780, SKOV3/PTX and A2780/PTX	miR-370-3p/RAB17 axis and CDK6/RB signaling pathway	Hsa_circ_0000714 was found to regulate RAB17 expression via sponging miR-370-3p, and through the CDK6/RB signaling pathway, so it showed to play a role in the malignant progression of the paclitaxel-resistant ovarian cancer cell A2780/PTX	[67]
circ_MUC16	30 pairs of tumor tissues and ANCTs/female BALB/c nude mice	A2780, SK-OV-3 and IOSE-80	miR-1182/S100B axis	∆ circ_MUC16: ↓ proliferation, glycolysis metabolism, migration and invasion ↑↑ circ_MUC16: ↓ effects of Propofol to promote the aggressive behaviors of ovarian cancer via sponging miR-1182	[68]
circ-FAM53B	54 pairs of tumor tissues and ANCTs	HO8910, SKOV3, OVCAR3, A2780 and IOSE80	miR-647, VAMP2 and MDM2	↑↑ circ-FAM53B: ↑ proliferation, migration, and invasion High levels of circ-FAM53B were correlated with clinical severity and poor prognosis of OC patients	[69]
circ-ABCB10	103 EOC tumor tissues and 53 EOC adjacent tissues as control	OVCAR3, UWB1.289, SKOV3, CAOV3 and IOSE80	miR-1271, miR-1252 and miR-203	High levels of circ-ABCB10 were associated with poor differentiation, large tumor size and advanced FIGO stage and worse OS in EOC patients ↑↑ circ-ABCB10: ↑ proliferation and ↓ apoptosis	[70]
circ-ABCB10	–	OVCAR3, UWB1.289, SKOV3, CAOV3 and IOSE80	miR-1271, Capn4/Wnt/β-catenin signaling	↑↑ circ-ABCB10: ↑ proliferation, invasion, Capn4/Wnt/β-catenin signaling pathway, ↓ apoptosis via sponging miR-1271	[71]
circ-CSPP1	12 borderline tumors, 117 ovarian carcinomas tissues, 12 benign ovarian tissues and 15 normal ovarian tissues	AOV3, A2780, OVCAR3	miR-1236-3p/ZEB1 axis	∆ circ-CSPP1: ↓ cell growth, migration, invasion, and EMT process High levels of circ-CSPP1 were associated with correlated with FIGO staging and differentiation	[72]
circ_0002711	54 pairs of tumor tissues and ANCTs/nude mice	SKOV3, OV90 and IOSE80	miR-1244/ROCK1 axis	∆ circ_0002711: ↓ cell viability, colony formation ability and aerobic glycolysis	[73]
circE2F2	–	OC tissues and cell lines	–	↑↑ circE2F2: ↑ proliferation, cell growth, metastasis, and glucose metabolism by stabilizing the E2F2 mRNA High levels of circE2F2 could strengthen the stability of the E2F2 mRNA through binding to the HuR protein High levels of circE2F2 were correlated with poor OS in OC patients	[74]
circHIPK2	46 pairs of tumor tissues and ANCTs/4–5-week-old female BALB/c-nude mice	SKOV3, A2780, SKOV3/DDP and A2780/DDP and IOSE80	miR-338-3p/CHTOP axis	∆ circHIPK2: ↓ cell proliferation, cell cycle entrance, migration and invasion in SKOV3/DDP and A2780/DDP cells and ↑ apoptosis and suppresses the 50% inhibitory concentration of DDP	[75]
circ_0000745	50 pairs of tumor tissues and ANCTs/4-week-old BALB/c nude mice	CoC1, ES-2, SW626, SK-OV-3 and IOSE-80	miR-3187-3p/ERBB4 axis and PI3K/AKT signaling pathway	∆ circ_0000745: ↓ proliferation, aggressiveness, EMT process, and stemness of SK-OV-3 cells	[76]

*∆* knock-down or deletion, *ANCTs* adjacent non-cancerous tissues, *EOC* epithelial ovarian cancer, *OS* overall survival, *EMT* epithelial-mesenchymal transition, *PFS* progression-free survival, *DFS* disease-free survival, *PTX* paclitaxel, *DDP* cisplatin, *HGSOC* high-grade serous ovarian cancer, *GTEx* genotype-tissue expression, *FIGO* International Federation of Gynecology and Obstetrics, *SOC* high-grade serous ovarian cancer

### Down-regulated circRNAs in ovarian cancer

A number of studies have reported down-regulation of certain circRNAs in ovarian cancer. For instance, circular RNA-ITCH has been shown to exert tumor suppressor role in this cancer. Down-regulation of circRNA-ITCH in this type of cancer has been associated with up-regulation of lncRNA HULC. Up-regulation of circRNA-ITCH has led to inhibition of cell proliferation, while up-regulation of HULC has resulted in opposite effects. Moreover, up-regulation of circRNA-ITCH has suppressed expression of HULC in these cells. While up-regulation of HULC has not affected expression of circRNA-ITCH, it has decraesed the inhibitory effect of circRNA-ITCH overexpression. Taken together, circRNA-ITCH can suppress proliferation of ovarian cancer cells through down-regulating HULC [77]. Moreover, circRNA-ITCH has been shown to suppress proliferation, invasiveness, and glycolysis of ovarian cancer cells through enhancing expression of CDH1 due to its sponging effect on miR-106a [78].

An RNA sequencing experiment has identified circPLEKHM3 as one of the utmost considerably down-regulated circRNAs in ovarian cancer samples versus normal tissues. Moreover, this circRNA has been found to be down-regulated in peritoneal metastatic ovarian cancers compared with primary cancers. Down-regulation of circPLEKHM3 has also been associated with poor prognosis. Mechanistically, up-regulation of circPLEKHM3 can inhibit cell growth, migration and epithelial-mesenchymal transition, while its silencing has led to opposite consequences. This circRNA acts through sponging miR-9 and regulation expressions of BRCA1, DNAJB6 and KLF4, and activity of AKT1 signaling. Moreover, the tumor-promoting effects of circPLEKHM3 silencing could be blocked by AKT inhibitor MK-2206 [79]. Another study has shown that the tumor suppressor role of curcumin in ovarian cancer is exerted through regulation of circ-PLEKHM3/miR-320a/SMG1 axis [80].

**Fig. 2:**
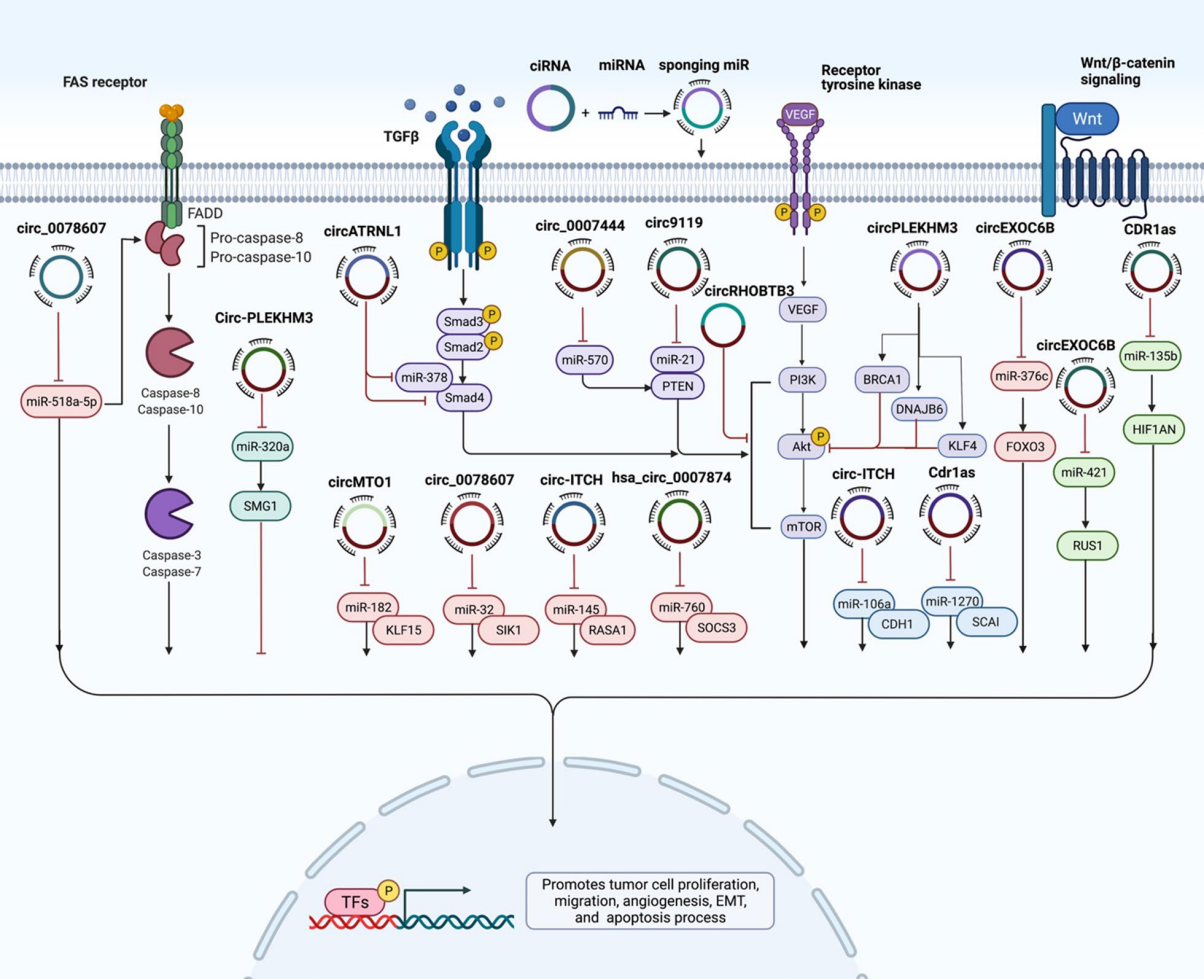
A schematic representation of the effects of some tumor suppressor circRNAs in the progression of ovarian cancer. The sponging effects of tumor suppressor circRNAs on oncogenic miRNAs such as miR-182, miR-32, miR-145 and miR-740 decrease proliferation and induce apoptosis of ovarian cancer cells. Thus, down-regulation of these circRNAs promotes progression of ovarian cancer

Hsa_circ_0078607 is another tumor suppressor circRNA whose inhibitory roles in ovarian cancer have been verified by different studies. This circRNA has been found to suppress progression of ovarian cancer through regulation of miR-518a-5p/Fas [81] and miR-32-5p/SIK1 [82] pathways. Moreover, down-regulation of this circRNA has predicted poor clinical outcome in high-grade serous ovarian cancer [83]. Figure 2 shows a number of tumor suppressor circRNAs in ovarian cancer.

CircEXOC6B is another tumor suppressor circRNA that inhibits proliferation and migratory potential of ovarian cancer cells and enhances their sensitivity to paclitaxel via modulation of miR-376c-3p/FOXO3 axis [84]. Moreover, it could progression of this cancer through influencing miR-421/RUS1 axis [85]. Notably, the tumor suppressor circRNA-9119 has been shown to affect miR-21-5p/PTEN/Akt axis [86]. Finally, circ-CDR1as could sequester miR-135b-5p to inhibit progression of ovarian cancer [87]. Moreover, it could up-regulate expression of SCAI to attenuate resistance of ovarian cancer cells to cisplatin through suppression of miR-1270 levels [88].

CircBNC2 is another tumor suppressor circRNA with potential biomarker role. It has been shown to perform better than HE4 and CA125 in differentiating patients with ovarian cancer from those with benign lesions or healthy subjects. Most notably, it could also separate early stage ovarian cancer from benign and healthy conditions. The performance of circBNC2 levels has been similar among pre- and postmenopausal subjects [89].

Table 2 shows the list of down-regulated circRNAs in ovarian cancer.

**Table 2:** Down-regulated circRNAs in ovarian cancer

circRNA	Clinical samples/animal model	Assessed cell lines	Targets/regulators/signaling pathways	Description	References
circPLEKHM3	5 tumor tissues and 5 normal ovarian tissues from patients with benign gynaecological diseases/4-week-old female athymic BALB/c nude mice	A2780, OV90 and MDAH2274	miR-9/BRCA1/DNAJB6/KLF4/AKT1 axis	↑↑ circPLEKHM3: ↓ cell growth, migration and EMT process Patients with low levels of circPLEKHM3 showed a worse prognosis	[79]
circPLEKHM3	35 pairs of tumor tissues and ANCTs/5-week-old female BALB/c athymic mice	SKOV3, A2780 and 293T cells	miR-320a/SMG1 axis	Curcumin treatment: ↑ circPLEKHM3 levels: ↓ cell proliferation and ↑ apoptosis	[80]
circ_100395	60 pairs of tumor tissues and ANCTs	A2780, OV2008, SKOV3, IGROV1 and ES-2	miR-1228	↑↑ circ_100395: ↓ tumor growth, metastasis and EMT process	[90]
circ_0078607	20 pairs of tumor tissues and ANCTs	SKOV3 and A2780	miR-518a-5p/Fas axis	↑↑ circ_0078607: ↓ proliferation and ↑ apoptosis via sponging miR-518a-5p	[81]
circ_0078607	43 pairs of tumor tissues and ANCTs/female BALB/c nude mice	HEY, ES-2 and IOSE80	miR-32-5p/SIK1 axis	↑↑ circ_0078607: ↓ proliferation, migration, invasion, and ↑ apoptosis via sponging miR-32-5p	[82]
circ_0078607	49 pairs of tumor tissues and ANCTs	–	–	Patients with low levels of circ_0078607 had advanced FIGO stage, higher serum CA125 level, shorter PFS and OS	[83]
circATRNL1	56 pairs of tumor tissues and ANCTs/6-week-old nude mice	A2780, SKOV3, CAOV‐3, SNU119 and IOSE80	miR-378/Smad4 axis	↑↑ circATRNL1: ↓ proliferation, invasion, migration, angiogenesis and ↑ apoptosis	[91]
circHIPK3	21 high grade EOC and 21 normal ovarian tissues	A2780, SKOV3 and IOSE80	–	∆ circHIPK3: ↑ proliferation, migration, and invasion and ↓ apoptosis	[92]
circRHOBTB3	–	ovarian cancer cells	PI3K/AKT signaling pathway	↑↑ circRHOBTB3: ↓ cell proliferation, metastasis and glycolysis via inactivating PI3K/AKT signaling pathway	[93]
circEXOC6B	60 ovarian cancer patients and 60 healthy controls/BALB/c mic	A2780, SKOV3 and IOSE-80	miR-376c-3p/FOXO3 axis	↑↑ circEXOC6B: ↓ proliferation, motility and chemoresistance of ovarian cancer cells to PTX via sponging miR-376c-3p Low levels of circEXOC6B were correlated with malignant pathological characteristics in ovarian cancer patients	[84]
circEXOC6B	–	A2870, SKOV3, OVCAR3 and IOSE80	miR-421/RUS1 axis	↑↑ circEXOC6B: ↓ proliferation, invasion and ↑ apoptosis via sponging miR-421	[85]
circ9119	40 ovarian cancer patients and 10 normal controls/5-week old female BALB/c nude mice	SKOV-3, HO-8910, A2780, ES-2, CAOV3, and OVCAR3 and FTE187	miR-21 and PTEN/Akt pathway	↑↑ circ9119: ↓ proliferation, viability and ↑ apoptosis	[86]
CDR1as	65 ovarian cancer patients and 37 normal controls	HO8910 and A2780	miR-135b-5p/HIF1AN axis	↑↑ CDR1as: ↓ proliferation, invasion and migration	[87]
Cdr1as	66 pairs of tumor tissues and ANCTs/4-week-old BALB/c female athymic mice	A2780, SKOV-3 and IOSE-80	miR-1270/SCAI axis	↑↑ CDR1as: ↓ proliferation and ↑ cisplatin-induced cell apoptosis in ovarian cancer cells via sponging miR-1270	[88]
circRNA1656	60 HGSOC tissues and 60 benign ovarian tissues	SKOV-3, HO 8910, A2780 and OVCAR-3	–	Downregulation of circRNA1656 was correlated with the FIGO stage of HGSOC	[94]
circ-ITCH	45 pairs of tumor tissues and ANCTs/5-week-old BALB/c nude mice	A2780, OVCAR3 and ISOE80	miR-106a/CDH1 axis	↑↑ circ-ITCH: ↓ proliferation, invasion, glycolysis and ↑ apoptosis via sponging miR-106a Levels of circ-ITCH were positively associated with 5-year OS of patients	[78]
circ-ITCH	75 pairs of tumor tissues and ANCTs	UWB1.289 and UWB1.289 + BRCA1	lncRNA HULC	↑↑ circ-ITCH: ↓ proliferation via downregulating HULC	[77]
circ-ITCH	–	SKOV3, A-2780, OVCAR-3, HO-8910 and IOSE80	miR-10a	↑↑ circ-ITCH: ↓ proliferation and ↑ apoptosis via sponging miR-10a	[95]
circ-ITCH	20 pairs of tumor tissues and ANCTs/6-weeks-old female BALB/c nude mice	SK-OV-3 and Caov-3	miR-145/RASA1 axis	↑↑ circ-ITCH: ↓ viability and motility by CCK8, cell cycle, wound healing assay and invasion via sponging miR-145	[96]
circ-ITCH	77 pairs of tumor tissues and ANCTs	SKOV3 and OVCAR-3	–	High levels of circ-ITCH were associated with small tumor size, decreased FIGO stage and prolonged OS ↑↑ circ-ITCH: ↓ proliferation and ↑ apoptosis	[97]
circBNC2	83 EOC patients, 83 benign ovarian cysts, and 83 healthy controls	–	–	CircBNC2 was found to be downregulated in EOC and could be promising novel biomarker for EOC	[89]
circMTO1	48 pairs of tumor tissues and ANCTs	SKOV3 and OVCAR3 and IOSE80	miR-182-5p/KLF15 axis	↑↑ circMTO1: ↓ proliferation and invasion	[98]
circ_0007444	87 pairs of tumor tissues and ANCTs/5-week-old female nude mice	SKOV3, OV420, A2780, CAOV3, OVCAR3 and HOSEpiC	miR-570-3p/PTEN axis	↑↑ circ_0007444: ↓ proliferation, migration, and invasion, and ↑ apoptosis via sponging miR-570-3p Low levels of circ_0007444 were correlated with advanced tumor stage and grade, large tumor size, and low 60-month percent survival	[99]
circLARP4	78 pairs of tumor tissues and ANCTs	–	–	Low levels of circLARP4 were correlated with FIGO stage, lymph node metastases and poor prognosis of OC patients	[100]
circLARP4	–	SKOV3, A2780, SW626, OVCAR3, OVCAR4 and HOSEpiC	miR-513b-5p/LARP4 axis	↑↑ circLARP4: ↑proliferation, invasion and migration	[101]
hsa_circ_0007874	4-week-old BALB/c nude mice	IGROV1, A2780, ES‐2, OV2008, and SKOV3 and ISOE80	miR-760/SOCS3 axis	↑↑ hsa_circ_0007874: ↓ proliferation and migration	[102]
circN4BP2L2	126 EOC patients, 126 benign ovarian cyst, and 126 healthy controls	SKOV3, OVCAR3, CAOV3, HO8910, TOV-112D, and IOSE80	–	↑↑ circN4BP2L2: ↓ migration and invasion Low levels of circN4BP2L2 were correlated with advanced tumor stage, worse histological grade, lymph node metastasis and distant metastasis in EOC	[103]

*∆* knock-down or deletion, *ANCTs* adjacent non-cancerous tissues, *EOC* epithelial ovarian cancer, *OS* overall survival, *EMT* epithelial-mesenchymal transition, *PFS* progression-free survival, *DFS* disease-free survival, *PTX* paclitaxel, *DDP* cisplatin, *HGSOC* high-grade serous ovarian cancer, *GTEx* genotype-tissue expression, *FIGO* International Federation of Gynecology and Obstetrics, *SOC* high-grade serous ovarian cancer

## Discussion

Ovarian cancer is a malignancy with highly variable clinical behavior ranging from good prognosis and high chance of cure to fast progression and poor clinical outcome [3]. This variable clinical manifestation most probably reflects dissimilarity in the biological characteristics of tumors [3]. Recent studies have used bioinformatics tools for identification of dysregulated genes in this kind of cancer to find the most important pathways, targets for treatment and candidate drugs [104].

CircRNAs with prominent roles in determination of cancer cells malignant behavior [105] and response to therapeutic options can explain at least some parts of this variability. These transcripts have critical roles in the regulation of expression of known tumor suppressor genes or oncogenes, since they can sequester miRNAs that suppress expression of these genes [106, 107].

CircRNAs have been shown to participate in the pathogenesis ovarian cancer through sponging miRNAs. CircMUC16/miR-199a-5p, circRNA_MYLK/miR-652, circRNA-UBAP2/miR-382-5p, circRNA-UBAP2/miR-144, circWHSC1/miR-145, circ_0013958/miR-637, circFGFR3/miR-29a-3p, hsa_circRNA_102958/miR-1205, circ_0072995/miR-147a, circ_0072995/miR-122-5p and circEPSTI1/miR-942 are examples circRNAs/miRNA axes in which an oncogenic circRNA acts as a sponge for a tumor suppressor miRNA. On the other hand, circPLEKHM3/miR-9, circPLEKHM3/miR-320a, circ_100395/miR-1228, circ_0078607/miR-518a-5p, circ_0078607/ miR-32-5p, circATRNL1/miR-378 and circEXOC6B/miR-376c-3p are examples of tumor suppressor circRNAs/oncogenic miRNA axes.

Since expression of circRNAs is influenced in the process of carcinogenesis and they are stable in the circulation of patients, circRNAs can act as diagnostic and prognostic markers in ovarian cancer. The former application is highlighted by the stability of these transcripts in the circulation of affected individuals which potentiates them as candidates for non-invasive methods of cancer detection. It is expected that therapeutic modalities affect expression of circRNAs, thus evaluation of expression of these transcripts in the peripheral blood might reveal response to therapy or tumor recurrence. Thus, they might replace the conventional nonspecific ovarian cancer biomarkers. Application of circRNAs as prognostic markers is supported by the studies that reported correlations between their levels and clinical as well as pathological parameters related to cancer prognosis. Future studies are needed to elaborate the association between expression levels of circRNAs and standard staging and grading systems of ovarian cancer. High throughput sequencing techniques would pave the way for identification of stage-/grade-specific panels of dysregulated circRNAs in ovarian cancer.

Moreover, circRNAs can affect response of ovarian cancer cells to paclitaxel. CircCELSR1, circ_CELSR1, circATL2, circNRIP1, circTNPO3 and hsa_circ_0000714 are examples of circRNAs that have important roles in either determination or modulation of chemoresistant phenotype. Since expression levels of these circRNAs affect responses of ovarian cancer cells to chemotherapy, they are putative markers that could be useful for monitoring molecular responses. Epithelial-mesenchymal transition of ovarian cancer cells has also been shown to be affected by a number of oncogenic circRNAs such as circ_100395, circFGFR3, circ_0000554, circCELSR1, circ-PTK2, circLNPEP, circ-CSPP1 and circ_0000745 as well as tumor suppressor ones such as circ_100395 and circPLEKHM3. The impact of non-coding RNAs on activity of cancer-related signaling is a crucial element in the carcinogenesis [108].

## Conclusion

Taken together, circRNAs can represent suitable candidate tumor markers in ovarian cancer and therapeutic targets to enhance response of cancer cells to conventional therapies. Moreover, results of in vitro and animal studies have proposed that targeting circRNAs can decrease malignant phenotype of ovarian cancer cells. A prominent limitation of studies conducted in this field is lack of verification of the obtained results in the clinical settings. Future studies are needed to verify these results in the clinical settings. Moreover, the importance of circRNAs in the determination of chemoresistance and possible targeted therapies for combating this phenotype should be assessed in future studies.

## Data Availability

The analyzed data sets generated during the study are available from the corresponding author on reasonable request.

## Abbreviations

circRNA: Circular RNA
miRNA: MicroRNA
ANCTs: Adjacent non-cancerous tissues
EOC: Epithelial ovarian cancer
OS: Overall survival
EMT: Epithelial-mesenchymal transition
PFS: Progression-free survival
DFS: Disease-free survival
PTX: Paclitaxel
DDP: Cisplatin
HGSOC: High-grade serous ovarian cancer
GTEx: Genotype-tissue expression
FIGO: International Federation of Gynecology and Obstetrics
SOC: High-grade serous ovarian cancer
